# Early-stage pregnancy recognition on microblogs: Machine learning and lexicon-based approaches

**DOI:** 10.1016/j.heliyon.2023.e20132

**Published:** 2023-09-14

**Authors:** Samer Muthana Sarsam, Ahmed Ibrahim Alzahrani, Hosam Al-Samarraie

**Affiliations:** a School of Strategy and Leadership, Coventry University, Coventry, United Kingdom; b Computer Science Department, Community College, King Saud University, Riyadh, 11437, Saudi Arabia; c School of Design, University of Leeds, Leeds, United Kingdom; d Centre for Instructional Technology and Multimedia, Universiti Sains Malaysia, Penang, Malaysia

**Keywords:** Sentiment analysis, Pregnancy recognition, Social media analysis, Emotions

## Abstract

Pregnancy carries high medical and psychosocial risks that could lead pregnant women to experience serious health consequences. Providing protective measures for pregnant women is one of the critical tasks during the pregnancy period. This study proposes an emotion-based mechanism to detect the early stage of pregnancy using real-time data from Twitter. Pregnancy-related emotions (e.g., anger, fear, sadness, joy, and surprise) and polarity (positive and negative) were extracted from users' tweets using NRC Affect Intensity Lexicon and SentiStrength techniques. Then, pregnancy-related terms were extracted and mapped with pregnancy-related sentiments using part-of-speech tagging and association rules mining techniques. The results showed that pregnancy tweets contained high positivity, as well as significant amounts of joy, sadness, and fear. The classification results demonstrated the possibility of using users’ sentiments for early-stage pregnancy recognition on microblogs. The proposed mechanism offers valuable insights to healthcare decision-makers, allowing them to develop a comprehensive understanding of users' health status based on social media posts.

## 1 Introduction

Pregnancy carries high medical and psychosocial risks that could lead pregnant women to serious health consequences. As per the World Health Organization (WHO) and medical standards, pregnancy is defined as the period of time during which a fetus develops inside a woman's uterus [1]. The Sustainable Development Goals (SDGs) and the WHO both have specific aims and address various challenges related to pregnancy issues. These include reducing the number of unintended pregnancies, improving maternal and child health outcomes through family planning education and services, and promoting gender equality. Empowering women to make informed decisions about their reproductive health and rights, and ensuring access to safe and effective family planning methods are also essential objectives in their efforts. In addition, the WHO has reported a number of challenges related to inadequate resources for monitoring health, which limits the reach and effectiveness of interventions. This includes the need to strengthen systems' capacity and infrastructure to provide comprehensive care to all pregnant women.

Monitoring changes in pregnant women's and infants' health is one of the critical tasks during the pregnancy period [2]. Several pregnancy-related health conditions, such as heartburn, cold, and body pain, may pose risks to the fetus [3]. Moreover, critical situations like unplanned pregnancy are associated with a higher likelihood of adverse exposures [4,5], increased maternal morbidity and mortality [6], preterm birth and low childhood weight [7,8], elevated risk of birth defects [9,10], and poorer maternal psychological health [11,12]. As the effective window for emergency contraception is about 120 h [13], and safe abortion access continues to be limited globally, delayed confirmation poses considerable risks to pregnant individuals [6]. Early, passive pregnancy recognition could increase the agency of a pregnant individual and speed up the adoption of pregnancy-safe behaviors, such as the avoidance of environmental risk factors [14], cessation of alcohol consumption or drug use [[15], [16], [17]], or provide the choice to discontinue a pregnancy at an earlier gestational age [18].

Early-stage recognition of pregnancy is extremely essential as a crucial step towards protecting women's and infants' lives. The development of automated tools for early pregnancy recognition has been limited by the low temporal resolution understanding of somatic changes in early pregnancy [19]. Also, the limited resources allocated for pregnancy monitoring programs impact the development of pregnancy recognition tools [20,21]. The literature revealed the application of various surveillance scenarios in the recognition and tracking of different health conditions [[22], [23], [24]]. Although a number of surveillance methods continue to rely on qualitative data, many are now adopting analytical approaches. The reason behind this lies in the technical innovation of surveillance technologies [25]. The use of advanced surveillance methods offers decision-makers numerous opportunities to take timely and effective actions, thus minimizing damage and averting widespread crises [26,27]. Our review of the literature showed limited evidence supporting the development of surveillance mechanisms for pregnancy recognition. Additionally, most of the proposed surveillance mechanisms for the recognition of pregnancy-related complications involve certain ubiquitous computing frameworks [28]. These frameworks are known to be costly and lack flexibility. Therefore, the objective of this study is to develop a lexicon-based method to provide effective real-time surveillance capable of recognizing early-stage pregnancy and associated risk factors on microblogs. The proposed mechanism extracts users' sentiments from their tweets and links them with pregnancy-related terms using part-of-speech tagging and association rules mining techniques. This recognition mechanism aims to assist healthcare decision-makers in better understanding the current health needs of pregnant women. Additionally, the proposed method offers an effective way to estimate the pregnancy rate (the proportion of pregnant females in a population) and assess associated risks.

## 2 Literature review

Many scholars have explored the potential of applying various methods to predict critical pregnancy events using cost-effective tools such as Twitter [29,30]. For instance, a study by Sarker et al. [31] examined the use of Twitter to discover cohorts of pregnant women, achieving a prediction performance of 84% using a rule-based approach. Golder et al. [32] developed an automatic classification system based on annotated tweets, achieving 88% accuracy in predicting pregnancy. Deep learning methods have also been employed for pregnancy recognition on Twitter. For example, Warikoo et al. [33] utilized an ensemble neural network model combining Long-short Term Memory (LSTM) – Recurrent Neural Networks (RNN) and Convolution Neural Networks (CNN), achieving an F1-score of 95%. Huang et al. (2017) developed a tree kernel-based model for categorizing pregnancy-related tweets with an accuracy of 84%. Chandrashekar et al. [3] explored the feasibility of using Twitter data for pregnancy recognition, achieving 81% accuracy with a Support Vector Machine (SVM) algorithm. Prieto et al. [34] examined the potential of social media platforms using regular expressions and NaiveBayes with Correlation-based Feature Selection (CFS) and achieved 90% accuracy.

Despite these advances, emotions associated with pregnancy have not been adequately considered in the existing pregnancy recognition mechanisms. During pregnancy, women experience both positive and negative emotions, such as happiness, worries, anxiety, and fears. Evidence from the literature shows that for many women, pregnancy involves a happy experience associated with positive expectations. However, at the same time, pregnancy also brings about worries and concerns [35]. In addition, a proportion of women suffer from psychological problems during pregnancy. Approximately 10–15% of women experience feelings of depression, and 3% develop post-traumatic stress disorder (PTSD) after giving birth [36]. Furthermore, the emotional state of a pregnant woman can influence the health of the infant. Previous research has shown that mothers with co-morbid anxiety and depression are more likely to have an infant with an insecure attachment [37]. These studies adopted the supervised learning technique, which has several limitations, including the difficulty in filtering health-related tweets' content and matching it with the examined topic [38]. Also, with the use of supervised methods, it is very unlikely that more than a few thousand tweets are relevant to a given discussion topic [38]. Still, the massive amount of data, in conjunction with the small amount of ground truth, poses a real challenge for the classification task [39,40]. Consequently, to overcome these challenges, a heuristic method using unsupervised learning techniques can deliver promising results in the health-related domain. For example, Lim et al. [41] applied an unsupervised learning method to textual data, together with temporal information, to identify latent infectious diseases in a specific location. Zhang and Elhadad [42] Presented a stepwise unsupervised method to recognize named entities from biomedical textual data. Meanwhile, the use of unsupervised learning methods via clustering algorithms might offer another means to assess clinical risk stratification [43]. Another implementation of clustering in the health discipline can be observed in learning laboratory tests and coded diagnoses [44].

The conclusion drawn from the previously mentioned studies highlights the potential of using various techniques, such as rule-based approaches, automatic classification systems, and deep learning methods, to achieve accurate pregnancy recognition. However, these approaches have largely overlooked the role of sentiments associated with pregnancy, despite evidence showing that pregnant women experience a wide range of emotions, including happiness, worries, anxiety, and fears. Moreover, previous studies have predominantly relied on supervised learning techniques, which have limitations in filtering and matching relevant health-related tweets, and they struggle to handle the massive amount of data with limited ground truth. Based on this, we decided to extract pregnancy-related sentiments as a step to detect early-stage pregnancy from Twitter data. Our hypothesis was that certain types of sentiments are related to pregnancy and can be used in the pregnancy recognition process. We aimed to answer the question: “What are the types of sentiments associated with pregnancy on Twitter?” To achieve this, we extracted pregnancy sentiments and terminologies from pregnancy-related tweets. Then, we utilized association rules mining to predict the frequent terminologies associated with early pregnancy. Finally, a predictive model was built to automatically predict the occurrence of pregnancy using the mapped features.

## 3 Study procedure

**Fig. 1:**
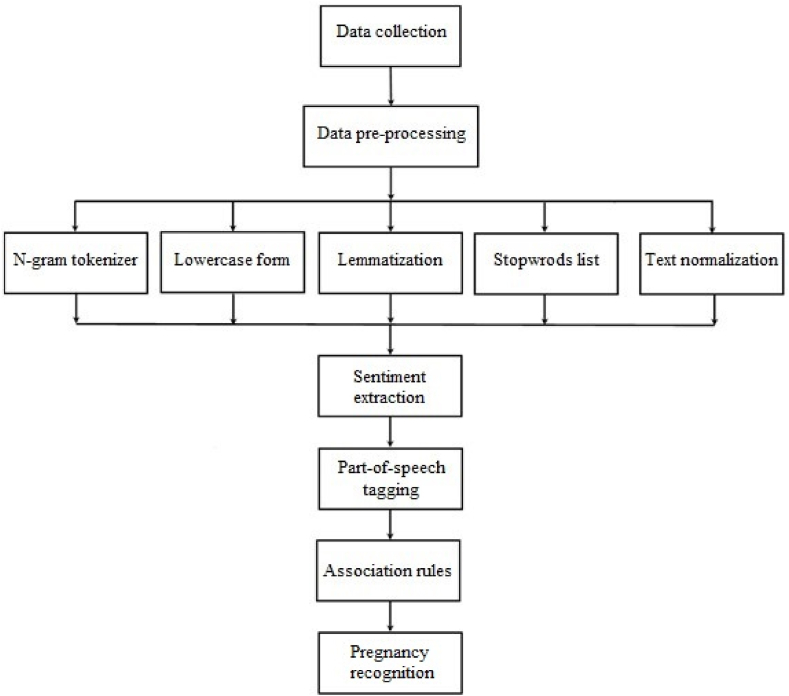
General procedure.

Fig. 1 summarizes the stages performed in this study, including data collection, data pre-processing, sentiment extraction, part-of-speech tagging, association rules mining, and pregnancy recognition. These stages are explained in detail in the following sections.

### 3.1 Data collection

A total of 1,738,759 English tweets were collected within a time span of six months (February 15th, 2019, till mid-August 2019). The data collection process was accomplished using the Twitter free streaming Application Programming Interface (API) based on the recommendation of Sarsam et al. [45]. The keywords used to obtain the desired tweets were ‘pregnancy’ and ‘non-pregnancy’. Subsequently, several data pre-processing procedures were implemented to improve the overall quality and reliability of the collected tweets.

### 3.2 Data pre-processing

At this stage, several preprocessing techniques were applied to process the collected data for future analysis. Data transformation was applied to the tweets to obtain a manageable representation since most popular machine learning algorithms face challenges in processing textual data smoothly. To do so, features of the retrieved data were extracted using the tokenization method via the n-gram tokenizer technique. After that, all the tokens were converted to lowercase form before applying the lemmatization technique. Lemmatization, in general, uses vocabulary and morphological analysis of words to remove inflectional endings and convert them to their dictionary form Balakrishnan et al. [46]. A stopwords list was applied to the lemmatized words, and then the length of each tweet was normalized using the L2 norm. As a result, a total of 753,558 tweets were analyzed after this data preparation process. Afterward, we engaged the expertise of two pregnancy specialists, each possessing 15 years of experience in the field, to evaluate the content of each tweet. Tweets related to pregnancy were classified as ‘Pregnancy’, while those unrelated to pregnancy were categorized as ‘Non-pregnancy’ tweets.

### 3.3 Sentiment extraction

At this stage, users' sentimental features were extracted from their tweets using the lexicon-based method. Therefore, users' emotions were extracted together with their polarity via the “NRC Affect Intensity Lexicon” [47] and SentiStrength [48,49], respectively.

NRC is a popular and robust approach for extracting users' emotions from their textual context [47,[50], [51], [52]]. In this sense, a list of English words and their associations was crafted to represent five basic emotions (anger, fear, sadness, joy, and surprise). According to Mohammad [47], For a given word and emotion X, the scores range from 0 to 1. Hence, a score of 1 means that the word conveys the highest amount of emotion X, while a score of 0 means that the word conveys the lowest amount of emotion X. The emotional features for each tweet were then calculated by adding the relevant associations of the words for a given lexicon (see section 3.2 for more details).

To extract the polarity from users' tweets, on the other hand, “SentiStrength” technique. Based on Culpeper et al. [53,54], for each tweet, this technique was used to assign scores ranging from ‘+1’ for ‘not positive’ to ‘+5’ for ‘extremely positive’ and ‘-1’ for ‘not negative’ to ‘-5’ for ‘extremely negative’. Based on these scores, we labeled the tweets with +5 as ‘Positive’ tweets and those with value −5 were labeled as ‘Negative’ tweets.

### 3.4 Part-of-speech tagging

It is essential to identify the main terms related to the examined categories (Pregnancy/Non-pregnancy). Therefore, the “Part-of-speech tagging” technique, a commonly used approach in social media analysis, was applied to identify the terms that can be used in different parts of speech [55]. Then, a Penn Treebank (PTB) tokenizer was applied to obtain words before using the probabilistic context-free grammar parser, based on the recommendation of Al-Samarraie et al. [56]. By doing so, we were able to extract only ‘noun’ words from a tweet to be analyzed with the association rules mining approach. Some of these nouns were used to form the terminologies of pregnancy symptoms. Finally, the relationship between such terminologies and the types of emotions in the tweet was established using the association rules technique. In other words, the extracted terms from this stage were mapped to each category using the association mining technique, which predicted category-related terms.

### 3.5 Association rules mining

To predict the terms that are highly related to each category (Pregnancy/Non-pregnancy), the association rules technique was applied. In this context, the Apriori algorithm was used to extract the hidden patterns in the data and establish meaningful relationships between the data features [57]. To do so, we configured the Apriori algorithm by setting the delta value at 0.05 to reduce the support until the minimum support is reached. The minimum metric score was set at 0.9, while the upper bound and lower bound support were set at 1.0. Then, we invoked this algorithm to extract category-related terms in association with category-related sentiments in the processed tweets. This helps in recognizing the pregnancy terms from those that are not about pregnancy by extracting pregnancy terms associated with pregnancy sentiments that frequently occur within the content of the data. For more information about the Apriori algorithm result, see Section 4.2.

### 3.6 Pregnancy recognition

At this stage, we created the training set (input) consisting of 753,558 tweets (i.e., after data pre-processing) with their sentimental features (anger, fear, sadness, joy, surprise, positive, and negative). Next, we utilized the Waikato Environment for Knowledge Analysis (Weka) to compare more than 15 classifiers on this data. For this purpose, the stratified tenfold cross-validation technique was employed to evaluate the overall learning process [58]. Finally, the top-three classifiers were selected, and their performance results are reported (for more information, see Section 4.3). These classifiers are NaiveBayes, DecisionTree, and Random Forest [59], J48 [60], and 1-rule classifier (OneR) [61]. For each classifier, we used the same settings (hyperparameters) as described in its source article.

To select the best classifier for predicting the category of the tweets (‘Pregnancy’ and ‘Non-pregnancy’), we used several evaluation metrics to examine the quality of the resulting predictions. These metrics include Accuracy, Kappa statistic, Root Mean Squared Error (RMSE), F1-score, and Confusion matrix. The classification results are summarized in Table 1.

**Table 1:** Classification results.

Classifier	Accuracy (%)	Kappa statistic (%)	RMSE (%)	F1-score (%)
*NaiveBayes*	91.24	79	11	100
*J48*	62.19	20	58	70
*OneR*	56.09	15	66	62

## 4 Results

### 4.1 Descriptive statistics results

**Fig. 2:**
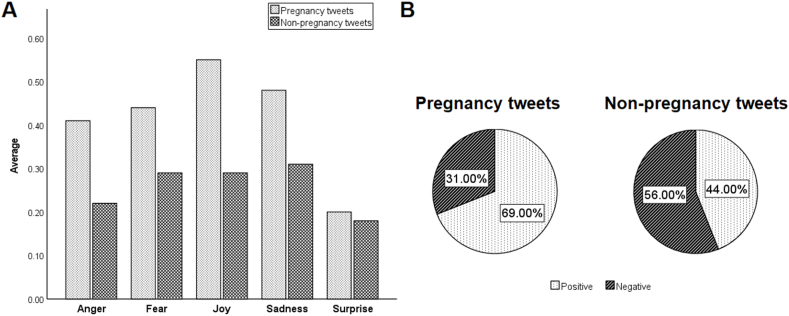
Sentiment analysis results. (A) Amounts of users' emotions in Pregnancy and Non-pregnancy categories; (B) Polarity results related to Pregnancy and Non-pregnancy categories.

To characterize the sentimental features obtained from the two types of tweets (pregnancy and non-pregnancy), we computed descriptive statistics and summarized the results in Fig. 2A. In this context, it was found that the amount of anger emotion (M = 0.41, SD = 0.05) in pregnancy-related statements is higher than in the non-pregnancy text category (M = 0.22, SD = 0.03). Additionally, the average of fear (M = 0.44, SD = 0.10) in pregnancy-related tweets is larger than in non-pregnancy-related tweets (M = 0.29, SD = 0.09). Our results also revealed that the amount of joy emotion is higher in pregnancy tweets (M = 0.55, SD = 0.14) than in the non-pregnancy category (M = 0.29, SD = 0.06). However, the amount of sadness in pregnancy texts (M = 0.48, SD = 0.26) is higher than the level of sadness in non-pregnancy-related tweets (M = 0.31).

Finally, the results illustrated that the amount of surprise in pregnancy-related statements (M = 0.20, SD = 0.08) is higher than that found in non-pregnancy tweets (M = 0.18, SD = 0.02). On the other hand, sentiment analysis polarity results (Fig. 2B) showed a larger percentage of positivity (69%) in pregnancy tweets compared to non-pregnancy tweets. In contrast, non-pregnancy tweets had a larger percentage of negativity (56%) compared to tweets related to pregnancy (31%). As a result, pregnancy tweets contained high positivity as well as high amounts of joy, sadness, and fear. However, fear, joy, and sadness sentiments were shown to be relatively higher than anger and surprise sentiments. To assess the similarities and differences between pregnancy and non-pregnancy statements, a *t*-test was utilized. The *t*-test results showed a significant difference (t = 2.89, p < 0.05) between the two groups.

### 4.2 Association rules

**Fig. 3:**
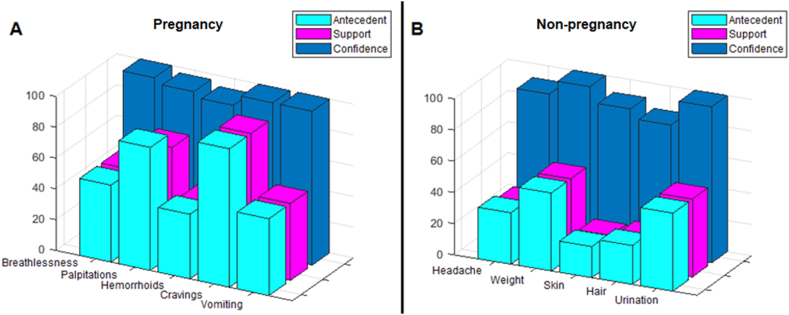
Association rules results. (A) Pregnancy terms; (B) Non-pregnancy terms.

The Apriori algorithm results are summarized in Fig. 3. Specifically, Fig. 3A revealed that only sadness and fear emotions were associated with pregnancy terms, such as Breathlessness, Palpitations, Hemorrhoids, Cravings, and Vomiting. Additionally, from Fig. 3B, it can be observed that anger emotion was found to be highly associated non-pregnancy-related terms: Headache, Weight, Skin, Hair, and Urination. From this, it can be concluded that there is a potential association between pregnancy and specific types of users' emotions within users' posts on social media platforms.

### 4.3 Classification results

Our classification results are summarized in Table 1, which showed that the NaiveBayes algorithm had the highest classification accuracy (91.24%), followed by J48 (62.19%) and OneR (56.09%) algorithms. Moreover, our results revealed that NaiveBayes achieved the highest kappa statistic value (79%) compared with J48 (20%) and OneR (15%). In contrast, the NaiveBayes algorithm produced the lowest RMSE value (11%) compared to J48 (58%) and OneR (66%). OneR also achieved the lowest F1-score value (62%), followed by J48 (70%) and NaiveBayes (100%), respectively.

**Fig. 4:**
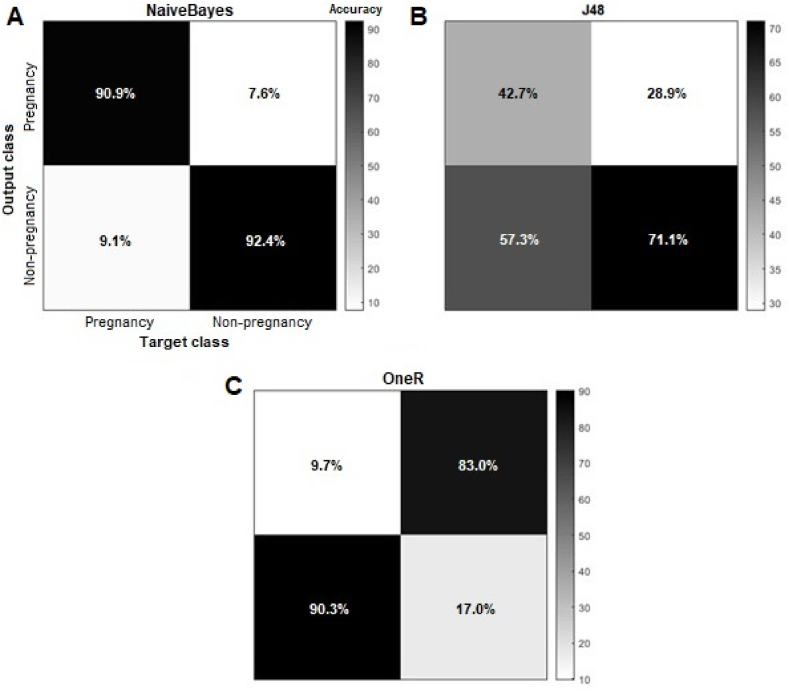
Confusion matrix results. (A) NaiveBayes confusion matrix; (B) J48 confusion matrix; and (C) OneR confusion matrix.

We also used the confusion matrix to assess the performance of the classification algorithms. The confusion matrix, in general, shows the capability of a classifier in identifying instances of different classes. It is usually utilized to measure the relationship between the predicted and the actual instances by representing instances along the diagonal of the confusion matrix. In our study, the confusion matrix results are shown in Fig. 4, where the value in every cell denotes the proportion of instances (tweets) that belong to each target class. In light of that, in Fig. 4A, the analysis of the confusion matrix's diagonal (90.9% and 92.4%) revealed that the NaiveBayes classifier had the best classification performance (compared with Fig. 4B and C) when predicting pregnancy and non-pregnancy tweets.

### 4.4 A comparison of the proposed approach with previous studies

To evaluate the robustness of our approach, we compared the obtained results with relevant studies from the literature. The comparison results are summarized in Table 2. The results showed that our method has a better performance than previous methods proposed in the literature. This leads us to assume that having emotions extracted from NRC Affect Intensity Lexicon can enhance the predictive capability of NaiveBayes classifier over other approaches applied in the literature.

**Table 2:** Pregnancy recognition techniques utilized in the literature.

No.	Study	Approach	Result (%)
1.	Golder et al. [32]	Developed automated classification system	F1-score = 88
2.	Warikoo et al. [33]	Ensemble Neural Network Model: LSTM; RNN and CNN. These ensemble representations were learned by SVM	F1-score = 95
3.	Sarker et al. [31]	Rule-based PIT	F1-score = 84
4.	Huang et al. [62]	SVM with tree kernel	• Accuracy = 84 • F1-score = 56
5.	Chandrashekar et al. [3]	SVM with RBF kernel	F1-score = 81
6.	Prieto et al. [34]	NaiveBayes and feature selection with CFS	F1-score = 90
7.	Our approach	NaiveBayes classifier and lexicon-based method	• Accuracy = 91.24 • F1-score = 100

### 4.5 Real-time evaluation

To further assess our method, we relied on social network analysis [63], where we predicted the network structure of both categories (Pregnancy and Non-pregnancy) using our original dataset. The tweets have been classified using NaiveBayes classifiers. Then, the tweets' information, including Twitter users and their activities, were used to plot the overall communication between the users within the same category. This advanced type of analysis demonstrates the tweets as a reflection of individuals’ activities (e.g., tweets by pregnant women) from the vertices (Twitter users) and edges (connections between these users in the form of Mentions, Replies to, and Tweets).

**Fig. 5:**
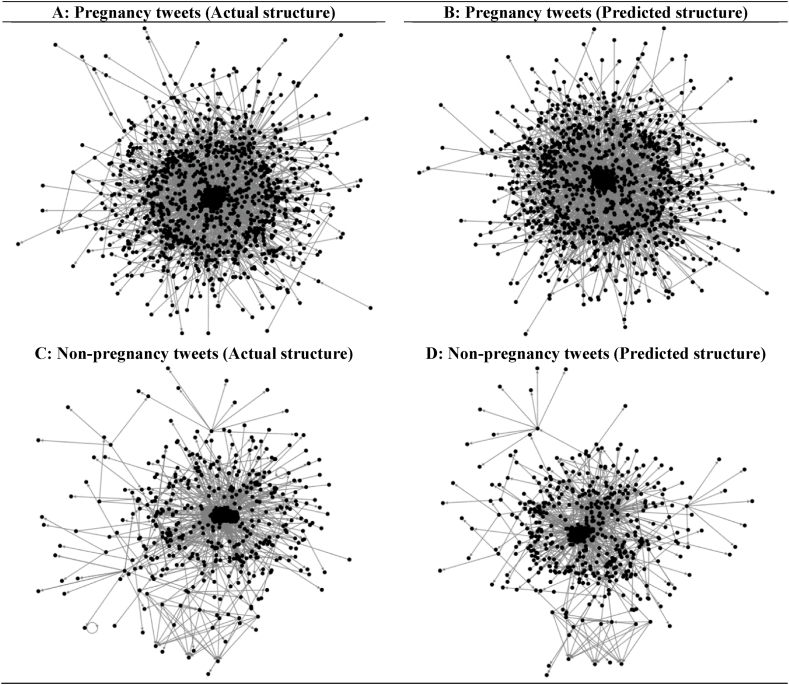
Real-time evaluation of the proposed approach using social network analysis. (A) Pregnancy tweets (Actual structure); (B) Pregnancy tweets (Predicted structure); (C) Non-pregnancy tweets (Actual structure); (D) Non-pregnancy tweets (Predicted structure).

Fig. 5A showed that the actual network structure for the pregnancy-related tweets was fairly similar to the predicted network structure of the pregnancy statements (Fig. 5B). Also, Fig. 5C showed that the actual network structure for non-pregnancy tweets was similar to the predicted network structure of the non-pregnancy tweets (Fig. 5D).

## 5 Discussion

Our results showed that pregnancy tweets contained high levels of positivity, joy, sadness, and fear. This finding appears reasonable because these emotions reflect interpersonal relationships between people and their social harmony with society. Additionally, it is expected to observe a high level of anger-related emotions in pregnancy since it is known from the pathogenesis literature as one of the most stressful reactions to pregnancy. Muscatello et al. [64] stated that anger is the most common negative emotion, and it plays a significant role in pregnancy. Previous scholars have highlighted that marijuana is the most commonly used illicit substance during pregnancy [65] in order to overcome their stress or depression [66], and thus the resulting anger [67,68]. In line with our results, a study by Tyrlik et al. [69] highlighted that some psychological dispositions and life conditions could lead mothers to experience negative emotions. As such, it can be aid that having a high level of fear-related emotions in our result is sensible as it is related to various mental and physical situations of the women before and after pregnancy [70]. Fear-related emotions during pregnancy can be also related to fear of childbirth which is a popular issue that affects women's wellbeing before and during pregnancy [71]. In this context, for some women, the fear mainly relates to childbirth, but for others fear occurs in association with anxiety [72]. However, for most women, pregnancy can bring both joy and excitement since it involves several personal changes, including physical, hormonal, psychological which affect the positive emotional experience of pregnant women [69]. Our results also showed the role of positive emotions in predicting early-stage pregnancy. Pregnancy is a happy experience associated with positive expectations [35] which may contribute to the high level of joy-related emotions in our results. Such emotions can be somehow related to the physical characteristics of a pregnant woman or due to having unexpected pregnancy. Consequently, our method highly contributes to the early pregnancy recognition process from social media websites. It also showed the worthiness of women's’ emotion in characterizing pregnancy-related issues.

## 6 Implications

The proposed method holds significant potential to contribute to the current development of surveillance systems aimed at rapidly identifying early health problems. By providing an intelligent mechanism for predicting early-stage pregnancy from Twitter data, our approach showcases the importance of emotions in detecting pregnancy-related characteristics, which are crucial for making informed decisions and evaluating outcomes in a specific population of women. The integration of emotions in our method offers valuable insights into the emotional well-being of pregnant individuals and can aid in understanding their psychological experiences during this period. Moreover, our method can function as a real-time surveillance system, empowering healthcare professionals to closely monitor the development of pregnancies and identify potential risk factors. The ability to detect early-stage pregnancy through social media data enables health practitioners to intervene promptly and provide appropriate support and care. This technology has the potential to revolutionize pregnancy monitoring and management, ensuring better health outcomes for both expectant mothers and their infants. Furthermore, we envision that our proposed technique can extend beyond gestation-related issues and be effectively applied in a broader context, encompassing various general health matters, including mental and physical states. By leveraging the power of social media data and emotion-based analysis, our method has the capacity to assist in monitoring public health trends and identifying health concerns on a larger scale.

## 7 Limitations and future works

This research does have some limitations that should be acknowledged. Firstly, our examination focused primarily on social media content related to pregnancy, which may not fully represent all aspects of pregnancy-related discussions. Expanding the scope to include a broader range of social media topics related to pregnancy could provide a more comprehensive understanding. Secondly, we solely obtained and analyzed English tweets since English is the most widely used language on Twitter. However, exploring data from other languages in future studies would be beneficial to assess the effectiveness of our method across diverse linguistic contexts. Thirdly, the current study utilized a limited number of keywords to search for tweets. Future research could incorporate additional and more specific keywords to capture a wider range of relevant tweets and sentiments. Fourthly, the Apriori algorithm was chosen for its efficiency in extracting meaningful patterns and building relationships between features. Although it served our purpose well, exploring alternative algorithms in future studies could validate the robustness of our findings. Finally, our analysis focused on five primary emotions (anger, fear, sadness, joy, and surprise) due to their prevalence in the literature. However, future researchers may consider investigating additional emotional categories and their potential associations with pregnancy, which could provide deeper insights into the emotional dynamics surrounding pregnancy discussions. Addressing these limitations in future studies will help strengthen the understanding and application of sentiment analysis and unsupervised learning techniques for pregnancy recognition and monitoring through social media data.

## 8 Conclusion

This study introduces an approach for recognizing early-stage pregnancy from Twitter messages. Users' sentiments were extracted using NRC Affect Intensity Lexicon and SentiStrength. Subsequently, pregnancy-related terms were identified and linked with pregnancy-related sentiments using part-of-speech tagging and association rules mining techniques. The findings demonstrated that pregnancy tweets exhibited high levels of positivity, along with significant occurrences of joy, sadness, and fear. Moreover, the classification results indicated the potential of utilizing users' sentiments for early-stage pregnancy recognition on microblogs. The proposed mechanism holds promise in providing healthcare decision-makers with valuable insights, empowering them to gain a comprehensive understanding of users' health status through the analysis of social media posts. This innovative approach has the potential to enhance early recognition and monitoring of pregnancy-related characteristics, thereby contributing to improved healthcare outcomes for pregnant individuals.

## Author contribution statement

SAMER MUTHANA SARSAM: Conceived and designed the experiments; Performed the experiments; Analyzed and interpreted the data; Contributed reagents, materials, analysis tools or data; Wrote the paper. Ahmed Ibrahim Alzahrani: Analyzed and interpreted the data; Contributed reagents, materials, analysis tools or data; Wrote the paper. Hosam Al-Samarraie: Performed the experiments; Analyzed and interpreted the data; Contributed reagents, materials, analysis tools or data; Wrote the paper.

## Funding

This work was funded by the Researchers Supporting Project number (RSP 2023 R/157), 10.13039/501100002383King Saud University, Riyadh, Saudi Arabia.

## Data availability statement

Data will be made available on request.

## Additional information

No additional information is available for this paper.

## Declaration of competing interest

The authors declare that they have no known competing financial interests or personal relationships that could have appeared to influence the work reported in this paper.

## References

[1] WHO (2023). https://www.who.int/news-room/questions-and-answers/item/pregnancy.

[2] Mcdonald C.R., Weckman A.M., Wright J.F.K., Conroy A.L., Kain K.C. (2020). Pregnant women in low-and middle-income countries require a special focus during the COVID-19 pandemic. Frontiers in Global Women's Health.

[3] Chandrashekar P.B., Magge A., Sarker A., Gonzalez G. (2017). Social media mining for identification and exploration of health-related information from pregnant women. arXiv preprint arXiv:1702.02261.

[4] Ranatunga I.D.J.C., Jayaratne K. (2020). Proportion of unplanned pregnancies, their determinants and health outcomes of women delivering at a teaching hospital in Sri Lanka. BMC Pregnancy Childbirth.

[5] Dott M., Rasmussen S.A., Hogue C.J., Reefhuis J. (2010). Association between pregnancy intention and reproductive-health related behaviors before and after pregnancy recognition, National Birth Defects Prevention Study. Matern. Child Health J..

[6] Allotey P., Ravindran T.S., Sathivelu V. (2021). Trends in abortion policies in low-and middle-income countries. Annu. Rev. Publ. Health.

[7] Rahman M.M. (2015). Is unwanted birth associated with child malnutrition in Bangladesh?. Int. Perspect. Sex. Reprod. Health..

[8] Shah P.S., Balkhair T., Ohlsson A., Beyene J., Scott F., Frick C. (2011). Intention to become pregnant and low birth weight and preterm birth: a systematic review. Matern. Child Health J..

[9] Zhang (2020). Clinical characteristics and fetal outcomes in women with epilepsy with planned and unplanned pregnancy: a retrospective study. Seizure.

[10] Wilson R.D. (2015). Pre-conception folic acid and multivitamin supplementation for the primary and secondary prevention of neural tube defects and other folic acid-sensitive congenital anomalies. J. Obstet. Gynaecol. Can..

[11] McCrory C., McNally S. (2013). The effect of pregnancy intention on maternal prenatal behaviours and parent and child health: results of an Irish cohort study. Paediatr. Perinat. Epidemiol..

[12] Qiu X., Zhang S., Sun X., Li H., Wang D. (2020). Unintended pregnancy and postpartum depression: a meta-analysis of cohort and case-control studies. J. Psychosom. Res..

[13] Upadhya K.K. (2019). Emergency contraception. Pediatrics.

[14] Kamai E.M., McElrath T.F., Ferguson K.K. (2019). Fetal growth in environmental epidemiology: mechanisms, limitations, and a review of associations with biomarkers of non-persistent chemical exposures during pregnancy. Environ. Health.

[15] Sundermann A.C. (2021). Week-by-week alcohol consumption in early pregnancy and spontaneous abortion risk: a prospective cohort study. Am. J. Obstet. Gynecol..

[16] Denny C.H., Acero C.S., Naimi T.S., Kim S.Y. (2019). Consumption of alcohol beverages and binge drinking among pregnant women aged 18–44 years—United States. MMWR (Morb. Mortal. Wkly. Rep.).

[17] Connery H.S., Albright B.B., Rodolico J.M. (2014). Adolescent substance use and unplanned pregnancy: strategies for risk reduction. Obstetrics and Gynecology Clinics.

[18] Shrestha D., Aryal S., Sharma B. (2018). Safety, efficacy and acceptability of early first trimester abortion using oral mifepristone and sublingual misoprostol. Journal of Nepal Health Research Council.

[19] Grant A., Smarr B. (2021).

[20] Shulman H.B., D'Angelo D.V., Harrison L., Smith R.A., Warner L. (2018). The pregnancy risk assessment monitoring system (PRAMS): overview of design and methodology. Am. J. Publ. Health.

[21] Bachiri M., Idri A., Fernández-Alemán J.L., Toval A. (2018). Evaluating the privacy policies of mobile personal health records for pregnancy monitoring. J. Med. Syst..

[22] Lansky A. (2007). Developing an HIV behavioral surveillance system for injecting drug users: the National HIV Behavioral Surveillance System. Publ. Health Rep..

[23] Mandyata C.B., Olowski L.K., Mutale W. (2017). Challenges of implementing the integrated disease surveillance and response strategy in Zambia: a health worker perspective. BMC Publ. Health.

[24] Kuehne A. (2019). Event-based surveillance at health facility and community level in low-income and middle-income countries: a systematic review. BMJ Glob. Health.

[25] Ribeiro-Navarrete S., Saura J.R., Palacios-Marqués D. (2021). Towards a new era of mass data collection: assessing pandemic surveillance technologies to preserve user privacy. Technol. Forecast. Soc. Change.

[26] Calvo R.A., Deterding S., Ryan R.M. (2020).

[27] Kitchin R. (2020). Civil liberties or public health, or civil liberties and public health? Using surveillance technologies to tackle the spread of COVID-19. Space Polity.

[28] Kazantsev A., Ponomareva J., Kazantsev P., Digilov R., Huang P. (2012). Proceedings of 2012 IEEE-EMBS International Conference on Biomedical and Health Informatics.

[29] Barnaghi P., Ghaffari P., Breslin J.G. (2016). 2016 IEEE Second International Conference on Big Data Computing Service and Applications (BigDataService).

[30] Klein A.Z., Cai H., Weissenbacher D., Levine L.D., Gonzalez-Hernandez G. (2020). A natural language processing pipeline to advance the use of Twitter data for digital epidemiology of adverse pregnancy outcomes. J. Biomed. Inf..

[31] Sarker A., Chandrashekar P., Magge A., Cai H., Klein A., Gonzalez G. (2017). Discovering cohorts of pregnant women from social media for safety surveillance and analysis. J. Med. Internet Res..

[32] Golder S. (2019). Pharmacoepidemiologic evaluation of birth defects from health-related postings in social media during pregnancy. Drug Saf..

[33] Warikoo N., Chang Y.-C., Dai H.-J., Hsu W.-L. (2018). Asia Information Retrieval Symposium.

[34] Prieto V.M., Matos S., Alvarez M., Cacheda F., Oliveira J.L. (2014). Twitter: a good place to detect health conditions. PLoS One.

[35] Røsand G.-M.B., Slinning K., Eberhard-Gran M., Røysamb E., Tambs K. (2011). Partner relationship satisfaction and maternal emotional distress in early pregnancy. BMC Publ. Health.

[36] Grekin R., O'Hara M.W. (2014). Prevalence and risk factors of postpartum posttraumatic stress disorder: a meta-analysis. Clin. Psychol. Rev..

[37] Schwartz D.A. (2020).

[38] Carvalho J.P., Rosa H., Brogueira G., Batista F. (2017). MISNIS: an intelligent platform for twitter topic mining. Expert Syst. Appl..

[39] Antoci A., Bonelli L., Paglieri F., Reggiani T., Sabatini F. (2019). Civility and trust in social media. J. Econ. Behav. Organ..

[40] Chowdhury S.M.H., Ghosh P., Abujar S., Afrin M.A., Hossain S.A. (2019). Emerging Technologies in Data Mining and Information Security.

[41] Lim S., Tucker C.S., Kumara S. (2017). An unsupervised machine learning model for discovering latent infectious diseases using social media data. J. Biomed. Inf..

[42] Zhang S., Elhadad N. (2013). Unsupervised biomedical named entity recognition: experiments with clinical and biological texts. J. Biomed. Inf..

[43] Huang Dong, Duan (2015). A probabilistic topic model for clinical risk stratification from electronic health records. J. Biomed. Inf..

[44] Poole S., Schroeder L.F., Shah N. (2016). An unsupervised learning method to identify reference intervals from a clinical database. J. Biomed. Inf..

[45] Sarsam S.M., Al-Samarraie H., Omar B. (2019). Proceedings of the 2019 8th International Conference on Software and Computer Applications.

[46] Balakrishnan V., Humaidi N., Lloyd-Yemoh E. (2016). Improving document relevancy using integrated language modeling techniques. Malays. J. Comput. Sci..

[47] Mohammad S.M. (2017). Word affect intensities. arXiv preprint arXiv:1704.08798.

[48] Sarsam S.M., Al-Samarraie H., Bahar N., Shibghatullah A.S., Eldenfria A., Al-Sa’Di A. (2021). International Conference on Human-Computer Interaction.

[49] Sarsam S.M., Al-Samarraie H. (2021). Early-stage detection of eye diseases on microblogs: glaucoma recognition. Int. J. Inf. Technol..

[50] Sarsam S.M., Al-Samarraie H., Ismail N., Zaqout F., Wright B. (2020). A real-time biosurveillance mechanism for early-stage disease detection from microblogs: a case study of interconnection between emotional and climatic factors related to migraine disease. NetMAHIB.

[51] Sarsam S.M., Al-Samarraie H., Al-Sadi A. (2020). Disease discovery-based emotion lexicon: a heuristic approach to characterise sicknesses in microblogs. Network Modeling Analysis in Health Informatics and Bioinformatics.

[52] Russo I. (2020).

[53] Culpeper J., Findlay A., Cortese B., Thelwall M. (2018). Measuring emotional temperatures in Shakespeare's drama. English Text Construction.

[54] Thelwall M. (2017). Cyberemotions.

[55] Ritter A., Etzioni O., Clark S. (2012). Proceedings of the 18th ACM SIGKDD International Conference on Knowledge Discovery and Data Mining.

[56] Al-Samarraie H., Sarsam S.M., A. I. J. C. i. H. B. Alzahrani (2023).

[57] Sarsam S.M., Al-Samarraie H. (2018). Towards incorporating personality into the design of an interface: a method for facilitating users' interaction with the display. User Model. User-Adapted Interact..

[58] Sarsam S.M. (2019). Proceedings of the 2019 8th International Conference on Software and Computer Applications.

[59] Georgen J., Pat L. (1995). Proc. Eleventh Conf. On Uncertainty in Artificial Intelligence.

[60] Salzberg S.L. (1994). C4. 5: programs for machine learning by j. ross quinlan. morgan kaufmann publishers, inc.. Mach. Learn..

[61] Holte R.C. (1993). Very simple classification rules perform well on most commonly used datasets. Mach. Learn..

[62] Huang (2017). Proceedings of the International Workshop on Digital Disease Detection Using Social Media 2017.

[63] Alzahrani A.I., Sarsam S.M., Al-Samarraie H., F. J. I. R. o. E. Alblehai, and Finance (2023). Exploring the sentimental features of rumor messages and investors. intentions to invest.

[64] Muscatello M.R.A. (2016). Anger in women treated with assisted reproductive technology (ART): effects on mother and newborn. J. Matern. Fetal Neonatal Med..

[65] McCabe J.E., Arndt S. (2012). Demographic and substance abuse trends among pregnant and non-pregnant women: eleven years of treatment admission data. Matern. Child Health J..

[66] Mark K., Desai A., Terplan M. (2016). Marijuana use and pregnancy: prevalence, associated characteristics, and birth outcomes. Arch. Wom. Ment. Health.

[67] Eiden R.D. (2011). Anger, hostility, and aggression as predictors of persistent smoking during pregnancy. J. Stud. Alcohol Drugs.

[68] Schuetze P., Lopez F.A., Granger D.A., Eiden R.D. (2008). The association between prenatal exposure to cigarettes and cortisol reactivity and regulation in 7‐month‐old infants. Dev. Psychobiol.: The Journal of the International Society for Developmental Psychobiology.

[69] Tyrlik M., Konecny S., Kukla L. (2013). Predictors of pregnancy-related emotions. J. Clin. Med. Res..

[70] Haines H.M., Rubertsson C., Pallant J.F., Hildingsson I. (2012). The influence of women's fear, attitudes and beliefs of childbirth on mode and experience of birth. BMC Pregnancy Childbirth.

[71] Nilsson C. (2018). Definitions, measurements and prevalence of fear of childbirth: a systematic review. BMC Pregnancy Childbirth.

[72] Handelzalts J.E. (2015). Personality, fear of childbirth and birth outcomes in nulliparous women. Arch. Gynecol. Obstet..

